# Chromosomal Integration of HHV-6 in a Preterm Neonate: A Rare Case of Hyperleukocytosis and Clinical Implications

**DOI:** 10.3390/pediatric16020037

**Published:** 2024-05-31

**Authors:** Palanikumar Balasundaram, Mohamed Sakr

**Affiliations:** 1 Division of Neonatology, Department of Pediatrics, Mercy Health—Javon Bea Hospital, Rockford, IL 61114, USA; 2 Division of Neonatology, The Children’s Hospital at Montefiore, Albert Einstein College of Medicine, Bronx, NY 10461, USA; msakr@montefiore.org

**Keywords:** hyperleukocytosis, neonates, human herpesvirus 6, chromosomally integrated HHV-6

## Abstract

Leukocytosis in neonates can occur because of infectious, inflammatory, malignant, or physiological processes. Hyperleukocytosis is defined as a total leukocyte count (TLC) exceeding 100,000 per mm^3^, warranting immediate evaluation. Neonates with hyperleukocytosis are at risk of leukostasis and the associated severe complications, including respiratory distress, myocardial ischemia, hyperuricemia, acute renal failure, infarction, and hemorrhage. Differentiating leukemia and leukemoid reactions in neonates presenting with elevated TLC is challenging but critical. We present a unique case of a preterm male neonate with hyperleukocytosis, initially suspected to have an underlying malignancy. The neonate’s clinical course was complicated by respiratory distress syndrome and anemia of prematurity, necessitating neonatal intensive care unit management. Further investigation revealed high human herpesvirus 6 (HHV-6) DNA levels in the whole blood, leading to a chromosomally integrated HHV-6 (ciHHV-6) diagnosis. CiHHV-6 is characterized by HHV-6 DNA integration into the host genome. Accurate diagnosis relies on whole-blood quantitative PCR, distinguishing ciHHV-6 from an active infection. The neonate remained asymptomatic, and antiviral treatment was deemed unnecessary. This case underscores the importance of recognizing ciHHV-6 as a potential cause of hyperleukocytosis in neonates and highlights the value of whole-blood PCR for differentiation. Understanding the spectrum of HHV-6 infection in neonates is vital for appropriate management and prognostication.

## 1. Introduction

Leukocytosis in neonates can manifest due to various underlying causes, including infectious, inflammatory, malignant, and physiological processes [1,2,3]. Leukocytosis becomes clinically significant when the total leukocyte count (TLC) exceeds 100,000 per mm^3^, a condition classified as hyperleukocytosis [2]. Hyperleukocytosis is relatively rare but requires prompt evaluation to exclude the possibility of leukemia or myeloproliferative disorders. Vigilant monitoring is crucial in neonates with hyperleukocytosis because of the potential complications, including respiratory failure, myocardial ischemia, hyperuricemia, acute renal failure, infarctions (splenic and brain), and hemorrhages (brain and lung) [3].

One challenging aspect in neonatology is distinguishing between leukemia and leukemoid reactions, which can contribute to an elevated TLC. A leukemoid reaction is diagnosed when the TLC exceeds 50,000 per mm^3^, and early mature neutrophil precursors are predominant. In contrast, leukemia is characterized by the predominance of immature neutrophils [4]. Leukemoid reactions can arise from various underlying illnesses, including infectious processes. This manuscript presents an exceptional case of a preterm male neonate who exhibited hyperleukocytosis and was subsequently diagnosed with chromosomally integrated human herpesvirus 6 (ciHHV-6). Understanding the concept of the chromosomal integration of HHV-6 into the human genome is essential for healthcare providers. This understanding enables the differentiation between ciHHV-6 and active HHV-6 infections, a crucial distinction that helps to avoid unnecessary antiviral treatment.

## 2. Case Presentation

A 26-week preterm male neonate was delivered via vaginal delivery to a 36-year-old mother with premature rupture of the membrane. The mother had cervical incompetence necessitating cervical cerclage and type 2 diabetes mellitus treated with metformin. Antenatal screening for group B streptococcus, hepatitis B virus, human immunodeficiency virus, syphilis, gonococcal, and chlamydia infections yielded negative results. Before delivery, the mother received betamethasone, magnesium sulfate, and antibiotics (ampicillin and azithromycin).

The neonate exhibited vigorous crying at birth and had Apgar scores of 5 and 8 at one and five minutes, respectively. He developed respiratory distress in the delivery room, necessitating continuous positive airway pressure (CPAP) support, and was later admitted to the neonatal intensive care unit (NICU). The surfactant was administered using the intubate–surfactant–extubate (IN-SUR-E) technique. Chest radiography revealed mild respiratory distress syndrome. Intravenous total parenteral nutrition, caffeine, and antibiotics (ampicillin and gentamicin) were initiated after obtaining a complete blood count (CBC) and blood culture.

**Figure 1:**
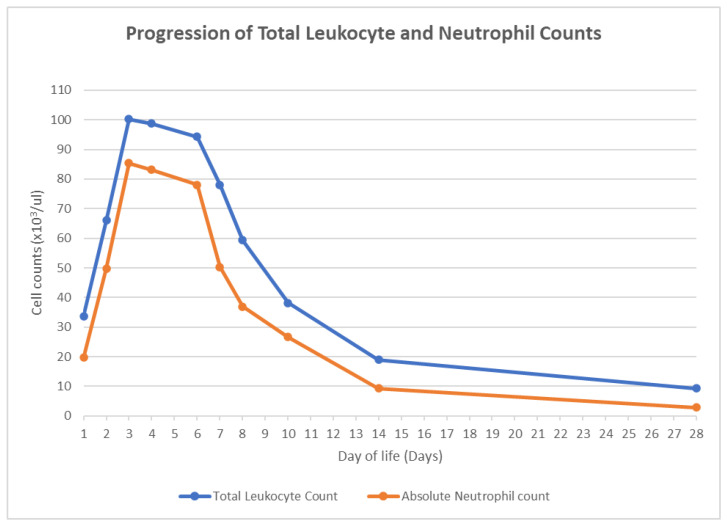
Progression of total leukocyte and neutrophil counts.

The initial CBC on admission revealed a TLC of 33,700 per microliter, comprising 59% neutrophils (N), 16% lymphocytes (L), 15% monocytes (M), and 5% bands (B). Subsequent CBCs showed an upward trend in TLC, reaching 66,100 per µL on day of life (DOL) 2 and 100,200 per µL on DOL 3 (Figure 1). A manual differential on DOL 3 showed 52% N, 7% L, 10% M, 8% B, 8% metamyelocytes, 3% promyelocytes, and 7% myelocytes. Remarkably, the neonate’s clinical condition was stable.

Due to hyperleukocytosis, the medical team conducted a repeat blood culture and lumbar puncture on DOL 4 and started a meningitis dose of ampicillin and cefepime. Acyclovir was added to cover the possibility of herpes simplex virus (HSV) infection. Cerebrospinal fluid (CSF) analysis revealed a glucose level of 59 mg/dL, protein level of 119 mg/dL, and no white blood cells. Blood and CSF cultures yielded negative results. The team discontinued antibiotics after two days and acyclovir after negative results for HSV 1 and 2 in the blood, CSF, and skin swabs. Qualitative polymerase chain reaction (PCR) for cytomegalovirus in the urine yielded negative results.

The CSF meningitis/encephalitis PCR assay was negative for all pathogens except human herpesvirus 6 (HHV-6). Subsequently, quantitative HHV-6 PCR was conducted on the same CSF sample, which showed 1600 copies/mL. The serum was analyzed for HHV-6-specific IgG antibodies via ELISA, and droplet digital PCR was used to quantify HHV-6 chromosomal integration. Plasma HHV-6 PCR revealed 470,000 copies/mL, while whole-blood HHV-6 PCR showed 5,500,000 copies of HHV-6 B DNA/mL. Serum IgG for HHV-6 was positive, whereas HHV-6 IgM was negative. The notably high number of copies of HHV-6 B DNA in the whole blood raised suspicion of chromosome-integrated HHV-6 (ci-HHV-6). Confirmatory chromosome integration testing in the whole blood demonstrated a ratio of HHV-6 B DNA copies to the number of nucleated cells of approximately 1 (0.96), consistent with the chromosomal integration of HHV-6 into the patient’s cell genome (Table 1).

Follow-up CBCs during the NICU stay indicated a decreasing TLC trend (Figure 1). No antiviral medications were initiated, as the diagnosis confirmed ciHHV-6B rather than an active infection. Additional prematurity-related morbidities, including bronchopulmonary dysplasia, apnea of prematurity, osteopenia of prematurity, and anemia of prematurity, were addressed during the NICU stay. Magnetic resonance imaging of the brain at term-corrected age showed no remarkable abnormalities.

The neonate remained clinically stable without febrile episodes throughout the NICU stay. On DOL 83, the neonate was discharged with iron and multivitamin supplements, and the infant’s growth was deemed appropriate. Given the elevated whole-blood HHV-6 levels, the neonate was scheduled for follow-up post-discharge to monitor for any evidence of acute illness, which is theoretically possible if he becomes immunosuppressed in the future.

## 3. Discussion

Chromosomally integrated HHV-6 (ciHHV-6) is a unique and often overlooked cause of transient hyperleukocytosis in neonates. ciHHV-6 refers to a condition where the entire HHV-6 genome becomes integrated into the host chromosome and is inherited with a Mendelian pattern. The preterm neonate in this case report demonstrated transient leukocytosis exceeding 100,000 per mm^3^, triggering a thorough diagnostic evaluation to exclude potentially life-threatening conditions. Subsequently, the investigation revealed an unforeseen diagnosis of ciHHV-6B as the root cause.

HHV-6 is a lymphotropic beta-herpesvirus with two types: HHV-6A and -6B [5]. The majority of primary infections are primarily caused by HHV-6B in infants. HHV-6 spreads through the saliva, infecting nearly all children by age three, and establishes latency with the potential for reactivation, especially in immunocompromised individuals [6]. Approximately 30% of children with a primary HHV-6B infection develop roseola infantum, marked by a 3–5-day fever followed by a morbilliform rash after the fever subsides and, occasionally, febrile seizures [7]. While HHV-6 reactivation is uncommon, it has been sporadically associated with conditions such as encephalitis, meningitis, pneumonitis, and retinitis [8].

Congenital HHV-6 infections in neonates primarily arise from two distinct mechanisms: transplacental transmission, accounting for 10%, and parental inheritance, comprising the remaining 90%. Although transplacental infections are less frequent, they contribute to neonatal HHV-6’s seropositivity. Another common mechanism involves chromosomally integrated HHV-6, in which the HHV-6 genome integrates into the telomeres of host chromosomes during latency. HHV-6 integration into gametes can result in the inheritance of HHV-6, a phenomenon commonly referred to as inherited chromosomally integrated HHV-6 (iciHHV-6). This phenomenon affects 1% of the population and is the primary mode of congenital HHV-6 transmission [9,10]. iciHHV-6 can occur with both HHV-6A and HHV-6B. ciHHV-6 inheritance follows a Mendelian pattern, with 50% of offspring inheriting ciHHV-6 [11].

Neonates born with iciHHV-6 carry HHV-6 DNA that is integrated into nearly every nucleated cell, resulting in high HHV-6 DNA levels. While plasma PCR is suitable for the detection of HHV-6 infection, whole-blood PCR is the gold standard for the diagnosis of inherited ciHHV-6. A quantitative PCR test in the whole blood detecting HHV-6 DNA greater than 1 × 106 copies/mL is indicative of iciHHV-6 [12]. However, samples like plasma, serum, and CSF contain fewer nucleated cells, leading to lower HHV-6 DNA counts released through cell lysis.

Plasma PCR tests cannot differentiate between ciHHV-6 and HHV-6 acquired through transplacental infection. If the plasma HHV-6 PCR level is elevated in neonates, it is imperative to conduct a whole-blood quantitative PCR test to exclude the possibility of ciHHV-6. ciHHV-6 is associated with significantly higher levels of viral DNA copies per milliliter (>5.5 log10 DNA copies/mL) in neonates compared to trans-placentally acquired HHV-6, which typically ranges from 3 to 3.5 log10 DNA copies/mL [13]. It is recommended that the PCR level be expressed as the viral DNA copies per cellular DNA. This provides valuable diagnostic insights, especially if the neonate exhibits leukocytosis or leukopenia.

Antibodies against HHV-6 glycoprotein B are detectable in over 50% of patients with HHV-6 infections but are present in only 10% of patients with ciHHV-6 [14]. However, they do not serve as a reliable means of distinguishing between ciHHV-6 and non-ciHHV-6 cases. The confirmatory tests for neonatal ciHHV-6 are ciHHV-6 screening among the neonate’s parents and/or the sequential testing of the neonate to determine the persistence of high viral DNA levels. Whole-genome sequencing (WGS) enables the early detection of ciHHV-6. Researchers using WGS on 7485 Japanese subjects identified integrated HHV-6 associated with polymorphisms on chromosome 22q [15]. Routine genomic screening in neonates with unexplained clinical conditions could reveal ciHHV-6 and other similar insertions, although it remains expensive to implement as a routine practice.

ciHHV-6 in neonates is generally benign, and substantial evidence linking it to major clinical complications is lacking. The likelihood of ciHHV-6 reactivation leading to an active HHV-6 infection is exceedingly low. Asymptomatic ciHHV-6 neonates typically do not require specific treatment. However, if a neonate with ciHHV-6 exhibits symptoms of meningoencephalitis, it is important to rule out cytomegalovirus and herpes simplex virus infections, and antiviral therapy may be considered. It is worth noting that active HHV-6 infections often do not respond well to acyclovir, and ganciclovir is usually recommended [16]. In cases of asymptomatic ciHHV-6, unnecessary antiviral treatment should be avoided.

## 4. Conclusions

The presented case of a preterm neonate with hyperleukocytosis ultimately led to an unexpected diagnosis of ciHHV-6 infection. This case underscores the importance of considering unusual etiologies of neonatal hyperleukocytosis and conducting appropriate diagnostic tests, including whole-blood quantitative PCR, to differentiate ciHHV-6 from active HHV-6 infection. Distinguishing between these entities is crucial in preventing unnecessary antiviral treatment and ensuring appropriate management. Given the unconventional methods that these viruses have adopted to endure within the human host, there remains a crucial need to explore the potential clinical ramifications of the integration of the viral genome with the human chromosome. Understanding and recognizing the intricacies of congenital HHV-6 infections, such as ciHHV-6, is pivotal for healthcare providers to ensure accurate diagnosis and appropriate management decisions.

## Figures and Tables

**Table 1:** Summary of HHV-6 laboratory test results.

Laboratory Test	Day of Life (DOL) Collected	Laboratory Value
CSF HHV-6 PCR	DOL 4	1600 copies/mL
Plasma/Serum HHV-6 PCR	DOL 7	470,000 copies/mL
Whole-Blood HHV-6 PCR	DOL 7	5,500,000 copies/mL
Whole-Blood HHV-6 DNA Copies to Nucleated Cell Count Ratio	DOL 7	0.96
Serum HHV-6 IgG	DOL 7	Positive
Serum HHV-6 IgM	DOL 7	Negative

## Data Availability

The original contributions presented in the study are included in the article, further inquiries can be directed to the corresponding author.
